# Bone-related behaviours of captive chimpanzees (*Pan troglodytes*) during two excavating experiments

**DOI:** 10.1007/s10329-022-01033-w

**Published:** 2022-11-19

**Authors:** Alba Motes-Rodrigo, Claudio Tennie, R. Adriana Hernandez-Aguilar

**Affiliations:** 1 Department of Early Prehistory and Quaternary Ecology, University of Tübingen, 72070 Tübingen, Germany; 2 Department of Ecology and Evolution, University of Lausanne, 1015 Lausanne, Switzerland; 3 Serra Húnter Program, Department of Social Psychology and Quantitative Psychology, University of Barcelona, 08035 Barcelona, Spain; 4 Centre for Ecological and Evolutionary Synthesis, University of Oslo, 0316 Oslo, Norway

**Keywords:** Bone tools, Tool-assisted excavation, Manual excavation, Innovation

## Abstract

After stone tools, bone tools are the most abundant artefact type in the Early Pleistocene archaeological record. That said, they are still relatively scarce, which limits our understanding of the behaviours that led to their production and use. Observations of extant primates constitute a unique source of behavioural data with which to construct hypotheses about the technological forms and repertoires exhibited by our hominin ancestors. We conducted two different experiments to investigate the behavioural responses of two groups of captive chimpanzees (*Pan troglodytes*; *n* = 33 and *n* = 9) to disarticulated, defleshed, ungulate bones while participating in a foraging task aimed at eliciting excavating behaviour. Each chimpanzee group was provided with bone specimens with different characteristics, and the two groups differed in their respective experience levels with excavating plant tools. We found that several individuals from the inexperienced group used the provided bones as tools during the task. In contrast, none of the individuals from the experienced group used bones as excavating tools, but instead continued using plant tools. These chimpanzees also performed non-excavating bone behaviours such as percussion and tool-assisted extraction of organic material from the medullary cavity. Our findings serve as a proof-of-concept that chimpanzees can be used to investigate spontaneous bone tool behaviours such as bone-assisted excavation. Furthermore, our results raise interesting questions regarding the role that bone characteristics, as well as previous tool-assisted excavating experience with other raw materials, might have in the expression of bone tool-assisted excavation.

**Supplementary Information:**

The online version contains supplementary material available at 10.1007/s10329-022-01033-w.

## Introduction

Early hominin technological artefacts found in the archaeological record include tools made from different raw materials, such as multiple rock types (e.g. basalt, flint, obsidian) and bone (reviewed by Toth and Schick 2015). Bone tools are often derived from ungulate bone fragments whose shape and surface texture have been anthropogenically modified. Such artefacts have been found in Early Pleistocene archaeological sites across the Old World (Backwell and d’Errico 2014) including Swartkrans, Drimolen, Sterkfontein, and Kromdraai, in South Africa (Robinson 1959; Brain and Sillent 1988; Backwell et al. 2008; Stammers et al. 2018), and Olduvai Gorge, in Tanzania (Leakey 1971; Shipman 1989; Backwell and d’Errico 2004; Pante et al. 2020). Bone tools have been attributed to early African hominins from the genera *Paranthropus* and *Homo*. Although the function(s) of these early bone tools remains contentious, most studies agree that they were probably used at least occasionally for digging activities that were likely related to foraging (Brain and Shipman 1993; Backwell and d’Errico 2001, 2005; Lesnik 2011).

Regardless of the precise task(s) for which early hominins employed bone tools, a necessary first step in the development of any technology—including bone technology—is the identification of a particular raw material as a potential tool source. Several hypotheses have been proposed to explain the emergence of such technological innovations (e.g. Fox et al. 1999, 2004; Gruber et al. 2016; Grund et al. 2019), all of which highlight the role that environmental factors play in the emergence of tool behaviours. The necessity hypothesis postulates that tool innovations are linked to caloric needs and thus often arise during periods of food scarcity where preferred, easily accessible food items become unavailable (Yamakoshi 1998; de Moura and Lee 2004). In such situations, individuals of certain species turn to tool use as a mean of accessing highly caloric but costly resources (i.e. in terms of foraging time and effort) such as encased foods [e.g. nuts (Boesch and Boesch 1990; Moura and Lee 2004)], insects protected by architectural structures (e.g. termites) or underground foods [e.g. honey (Gruber et al. 2016)]. The opportunity hypothesis postulates that tool use emerges as a consequence of chance encounters with, for example, potential raw materials that are suitable for tool manufacture (Fox et al. 2004; Koops et al. 2013). A third hypothesis, the relative profitability hypothesis, stipulates that tool use emerges as a response to a caloric need when using tools is more efficient than not using tools (Rutz and St Clair 2012). Varying degrees of support have been found for these hypotheses as explanations of how and why some populations of nonhuman great apes (henceforth apes) develop or do not develop specific tool-using behaviours (Fox et al. 2004; Gruber et al. 2016; Koops et al. 2013; Sanz and Morgan 2013). If apes are assumed to represent valid phylogenetic models for early hominin behaviour (Wynn et al. 2011; Carvalho and McGrew 2012; Rolian and Carvalho 2017), then similar ecological pressures to those described for apes could have played a role in the initial emergence and/or maintenance of early hominin bone technologies.

To date, two experimental studies have tested the use of skeletal materials as tools by nonhuman primates. Westergaard and Suomi (1994) investigated whether nine captive tufted capuchin monkeys (*Sapajus apella*, formerly *Cebus apella*) could, in the absence of demonstrations, use bovid limb fragments as pounding tools in order to crack open nuts and/or as cutting tools to open a puzzle box baited with food. The authors found that three monkeys out of the nine tested performed both tasks. Roffman et al. (2015) assessed whether bonobos (*Pan paniscus*) would use cervid antlers (and other raw materials) as excavating tools in the absence of demonstrations. The authors found that captive bonobos from two different groups employed the antlers as well as plant tools to excavate for buried food items.

Currently, the spontaneous bone tool-using abilities of chimpanzees (*Pan troglodytes*)—one of the closest living relatives of modern humans and the non-human primate species with the broadest tool repertoire in the animal kingdom—remain unknown. Chimpanzees use a wide variety of tools flexibly, as well as select appropriate tools for specific tasks, thus showing an understanding of tool material properties (Sakura and Matsuzawa 1991; Manrique et al. 2010; Sirianni et al. 2015; Lamon et al. 2018). However, to the best of our knowledge, no study to date has investigated whether untrained captive chimpanzees can identify and use bones as tools—a skill that must have necessarily preceded the emergence of bone technologies in our lineage. More generally, chimpanzees in captivity have rarely been presented with disarticulated bones. Wynn and McGrew (1989, p. 387), citing Kithara-Frisch et al. (1987), reported that “chimpanzees shown how to use hammer-stones to smash long-bones then used the resulting bone fragments to puncture a skin covering a bottle of sweetened drink”. In a later bone-related study, Pickering and Wallis (1997) provided four groups of captive chimpanzees with the disarticulated bones of bovids and cervids, which were cleaned of adhering soft tissues and then coated with foods found palatable by the subjects. The goal of the study was to investigate the bone surface modifications inflicted by the teeth of chimpanzees as they gnawed and chewed the bone coatings. In the wild, chimpanzees hunt a variety of small and medium-sized vertebrates (reviewed by Newton-Fisher 2007), sometimes with the assistance of wooden tools (Pruetz and Bertolani 2007) and occasionally create bone assemblages (Plummer and Stanford 2000). In addition, wild chimpanzees sometimes break large bones in order to access the marrow (Goodall 1986) and have also been reported to consume bone marrow with the aid of plant tools (Boesch and Boesch 1990; Sanz and Morgan 2007). However, there are no reports of the use of bones as tools by wild chimpanzees.

Despite the absence of bone tools in the repertoire of wild chimpanzees, investigating bone-related behaviours in this species can provide important insights into their innovative and tool-using abilities. Broadly, experiments in captivity that explore behaviours not yet described in wild populations provide valuable data on the potential form in which these behaviours could be expressed in the wild (see also van Lawick-Goodall 1971). Furthermore, experiments on captive animals that explore the acquisition of novel tool behaviours allow assessing the possibility that these behaviours would be eventually observed or innovated in wild populations. Indeed, such possibility has already taken place multiple times. Kohler (1925) described spontaneous ant-dipping, fluid-dipping and tool excavation in captive chimpanzees decades before these behaviours were first reported and described in wild chimpanzee populations (Goodall 1986; McGrew 2021; Hernandez-Aguilar et al. 2007). Visalberghi (1987) described spontaneous nut-cracking using stone hammers in captive tufted capuchins before reports of this behaviour from wild populations of the closely related bearded capuchins (*Sapajus libidinosus*, formerly *Cebus libidinosus*) were published (Fragaszy et al. 2004a, b; de Moura and Lee 2004). Outside of the primate lineage, Goffin cockatoos (*Cacatua goffiniana*) were reported to manufacture and use tools in captive settings (Auersperg et al. 2016) years before this behaviour was discovered in wild individuals (O’Hara et al. 2021). Similar to the present study, previous work has been conducted investigating the abilities of captive great apes to innovate behaviours absent in wild conspecifics. For instance, Bandini et al. (2021) reported the spontaneous innovation of nut-cracking using wooden hammers in captive orangutans, whereas Neufuss et al. (2017) described nut-cracking with stone hammers in captive bonobos.

In this study we focused on a behaviour that some authors have deemed crucial for the evolution of our species, namely tool-assisted excavation of edible underground resources (Laden and Wrangham 2005). Tool-assisted excavation during foraging has been described in both wild (Hernandez-Aguilar et al. 2007; Estienne et al. 2017; McLennan et al. 2019) and captive (Kohler 1925; Motes-Rodrigo et al. 2019) chimpanzees, as well as in bearded capuchin monkeys (de Moura and Lee 2004; Falótico et al. 2017). In our lineage, this behaviour is present in modern humans (Lee 1979), and in the past might have involved the use of bone tools (Brain and Shipman 1993; see above). In order to investigate whether chimpanzees would recognize and use bones as excavating tools, we conducted two independent exploratory experiments with two captive populations of chimpanzees that varied in their degree of excavating experience, tool use proficiency, raw material availability and group size. Each population was provided with several defleshed, disarticulated bones or fragments that could be used as excavating tools to obtain buried food items. The aims of our experiments were (1) to describe the behavioural responses of captive chimpanzees to disarticulated bones, (2) to assess whether chimpanzees could identify (and use) bones as tools in order to excavate, and (3) to describe the excavating actions performed by the chimpanzees using the bones as tools.

## Materials and methods

### Study sites and subjects

We studied two groups of captive chimpanzees housed at two different zoological institutions. The first group of chimpanzees is housed at Kristiansand Zoo, Norway, and consists of nine individuals (four adult males, four adult females and one 5-year-old juvenile female) that ranged in age from 6 to 43 years at the time of testing. All the chimpanzees were born in captivity, and all but one of the chimpanzees were reared by their mothers. The chimpanzees have access to both an indoor (135 m^2^) and an outdoor (1840 m^2^) enclosure, as well as to individual sleeping rooms. The outdoor enclosure is a forested island with natural soil, rocks, and vegetation, surrounded by a water-filled moat. Bedding material and structural enrichment such as logs, ropes, platforms and climbing structures are present both in the indoor and outdoor enclosures. In addition, several devices aimed at promoting the use of tools, such as a puzzle box (i.e. nut maze) and several foraging enrichment devices (i.e. artificial termite mound and perforated hanging log) were available in the indoor enclosure and baited regularly with food. The chimpanzees were also often provided with other enrichment elements such as hose fragments, tubes and plastic bottles baited with preferred foods such as honey, yogurt and fruit smoothies. These preferred foods could be obtained using tools that the chimpanzees manufactured from leafy branches provided every day in the indoor enclosure. Consequently, the chimpanzees at Kristiansand Zoo manufactured and used plant tools on a daily basis in order to forage both from the fixed and mobile enrichment devices.

The second chimpanzee group is housed in Leintal Zoo in Schwaigern, Germany, and consists of 33 chimpanzees (16 males, 17 females) that ranged in age from 5 to 47 years (mean ± SD, 22.4 ± 1.58 years) at the time of testing. All but one of the chimpanzees were born in captivity, and 25 of the chimpanzees were mother reared. The eight hand-reared individuals were taken care of by the zoo staff either after being abandoned as infants at the zoo by previous owners (one chimpanzee) or after being rejected by their mothers (seven chimpanzees). All hand-reared individuals were successfully reintroduced into the chimpanzee group as juveniles. The chimpanzees have access to an indoor (329.82 m^2^) and an outdoor enclosure (958.25 m^2^) and several sleeping rooms out of sight of zoo visitors. Bedding material and structural enrichment such as logs, ropes, platforms and climbing structures were present both in the indoor and outdoor enclosures. The outdoor enclosure consists of four interconnected areas surrounded by a 4-m-high metallic mesh that delimits the enclosure from the sides and from the top, where it acts as a roof. The ground of the outdoor enclosure is natural dirt. No woody vegetation is present in the outdoor enclosure, although the chimpanzees occasionally acquire sticks and branches from the vegetation surrounding the enclosure by pulling them through the mesh. The tool use repertoire of the chimpanzees was limited to using sticks to attract the attention of the keepers (i.e. poke them) and to rake food closer to the fence when food was placed in the vicinity of the outdoor enclosure during feedings. The chimpanzees were often provided with scattered seeds and nuts in the outdoor enclosure as foraging enrichment.

The chimpanzees at Kristiansand Zoo had participated in an excavating experiment 3 years before the present study, when buried food items, as well as potential plant tools, were presented to the group in their outdoor enclosure [for details, see experiment 1 in Motes-Rodrigo et al. (2019)]. Thus, the chimpanzees at Kristiansand Zoo were familiar with excavating tools, and all but one chimpanzee had previously engaged in tool-assisted excavation (Motes-Rodrigo et al. 2019). Prior to the start of the current experiments, the chimpanzee keepers at both institutions confirmed that none of the chimpanzees had any previous experience with bones, including disarticulated and defleshed bones. The chimpanzee keepers at Leintal Zoo were also interviewed regarding the previous experience or exposure of the chimpanzees to excavation of any kind. These keepers reported that some of the chimpanzees (it was not noted which of them) had very limited experience with underground items (e.g. insects, baited buried bottle). Detailed descriptions of these reports can be found in the supplementary material. The keepers had no recollection of the chimpanzees witnessing any gardening activities involving excavation at the zoos, although this cannot be completely discounted as a possibility.

### Experimental design

Data were collected at Kristiansand Zoo during May and June 2016 on 19 non-consecutive days (based on weather conditions) and at Leintal Zoo from 3 to 10 October 2018 on 7 consecutive days. Three experimental conditions were implemented in each zoo following the protocol described by Motes-Rodrigo et al. (2019). Before the first experimental condition, the experimenter dug five holes in a study area of the outdoor enclosure chosen to maximize visibility while the chimpanzees were in the indoor enclosure and were thus unable to observe the experimenter. The holes were 30 cm deep and 15 cm in diameter, following the dimensions of holes excavated by wild chimpanzees in Issa, Tanzania (Hernandez-Aguilar et al. 2007). In the case of the Kristiansand chimpanzees, the study area of the present experiment was at a different location in the outdoor enclosure from the study area where the previous excavating experiment had taken place 3 years prior (Motes-Rodrigo et al. 2019). Furthermore, each hole at Kristiansand Zoo was marked with a flag composed of a yellow piece of paper glued to a wooden skewer to facilitate its detectability (Motes-Rodrigo et al. 2019). This measure was not implemented at Leintal because detectability of the holes was not an issue due to the absence of vegetation in the study area.

The first experimental condition (Open holes condition) comprised two sessions at Kristiansand Zoo and, due to time constraints, one session at Leintal Zoo. During the Open holes condition, the holes were left uncovered and a whole, previously washed food reward was placed inside each hole (grapes at Kristiansand and apples at Leintal). The aim of the Open holes condition was to attract the Kristiansand chimpanzees to the study area [as they were already familiar with buried items; see experiment 1 in Motes-Rodrigo et al. (2019)] and to familiarize the Leintal chimpanzees with the presence of food within the holes in the study area. The second experimental condition (Loose soil condition) took place over two sessions at Kristiansand Zoo and one session at Leintal Zoo. For the Loose soil condition, we placed a food reward inside each hole, but this time covered each hole with natural loose soil from the outdoor enclosure. The aim of this condition was to remind the Kristiansand chimpanzees of the testing procedure and to familiarize the Leintal chimpanzees with the presence of food hidden under soil. In sum, the two first conditions constituted a phase of familiarization for the chimpanzees with the experimental set-up and we did not expect frequent tool use as the rewards were easily accessible by hand. In Kristiansand Zoo the experimenter could only access the study area to rebait the holes  in the morning before the chimpanzees left their sleeping quarters. Thus, filming only took place for 2–3 h each day, although the chimpanzees could access the study area all day long. In Leintal Zoo, the sessions of the Open holes condition and the Loose soil condition lasted 2 h each (9–11 a.m.) as the chimpanzees could be moved out of the study area by the keepers during the day allowing the the experimenter to rebait the holes.

After the familiarization phase, we conducted the Compacted soil condition for a total of 15 days at Kristiansand Zoo and for 6 days at Leintal Zoo. During the Compacted soil condition, a fruit reward was placed inside each of the holes in the study area and each hole was then filled with compacted natural soil from the outdoor enclosure. Experimenters compacted the soil without the use of tools by first tamping it by hand and then stomping on it.

At Kristiansand Zoo, we conducted one session per day starting when the chimpanzees were allowed into the outdoor enclosure (approximately 8:30 a.m.) and ending 2–3 h later. The total testing time at Kristiansand Zoo was approximately 35 h. Holes were reshaped to their original dimensions after each session. At Leintal Zoo, two sessions were conducted each day (total *n* = 12), with the morning session starting at 9 a.m. and the afternoon session starting around 12 p.m. Sessions lasted between 1 and 5 h depending on the daily routines of the chimpanzee keepers. Unfortunately, the recordings of session 5 were lost due a technical problem with the video camera, and therefore only 11 sessions from Leintal Zoo were included in the analysis. The total testing time at Leintal Zoo was 30 h.

During the Compacted soil condition, the Kristiansand Zoo chimpanzees were provided with a total of 19 disarticulated and defleshed horse (*Equus caballus*) bone fragments that could be used as excavating tools (see detailed measurements in Table S1). Bones were obtained from horse carcasses that were donated to the zoo to feed its carnivores. In order to standardize the bone specimens that were then presented to the chimpanzees, we sawed the epiphyses from several horse humeri and femora and then, for some specimens, split their remaining diaphyses longitudinally with a saw. This standardization process was conducted to replicate as closely as possible the bone tools found in the early hominin archaeological record (e.g. those from Swartkrans and Drimolen), which are mainly long limb bone shaft fragments without their complete original circumference and without epiphyses. This process also allowed for easier cleaning of specimens prior to their introduction into the chimpanzee enclosures and facilitated the measurement of potential quantitative changes in bone dimensions due to their use by the  chimpanzees. The starting bone fragment (*n* = 19) dimensions were as follows: mean superoinferior length ± SD, 16.0 ± 4.28 cm; mean maximum width ± SD, 6.22 ± 0.93 cm; mean weight ± SD, 308.94 ± 97.58 g. The bone fragments were provided to the chimpanzees by placing them in the study area next to the covered holes, flat on the ground.

The Kristiansand chimpanzees were provided with one to three horse bone specimens at a time, and they had access to each bone for a period ranging from 1 to 5 days (see Fig. S1, Online Resource). This variation was due to the fact that, although we aimed to recover each fragment after 24 h, the chimpanzees often hid/carried the bones with them between enclosures, preventing the experimenter from recovering them. Furthermore, seven of the bone fragments provided were lost, presumably in the water-filled moat or hidden in the vegetation.

**Fig. 1:**
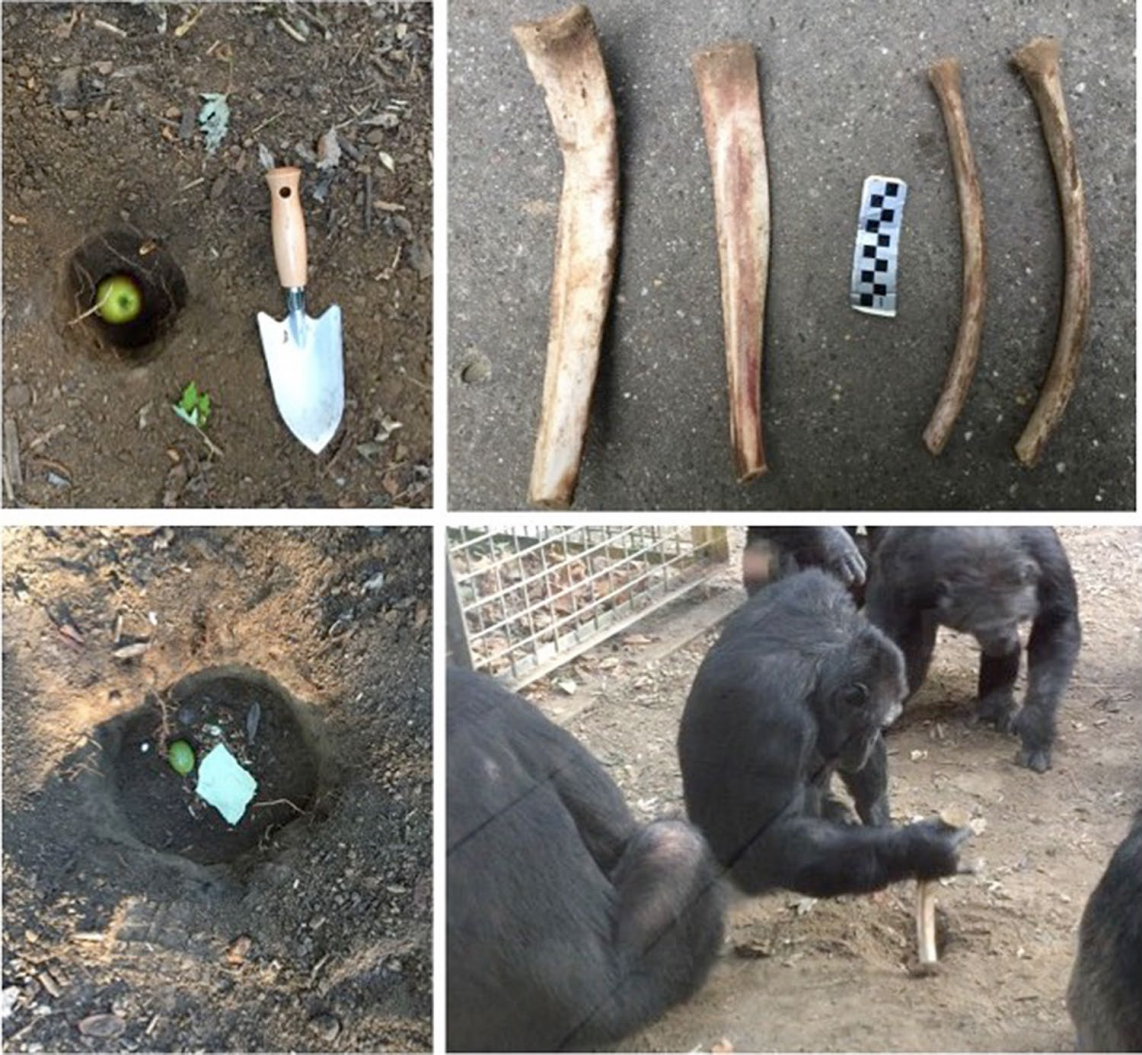
*Top left* Baited hole in the Open holes condition.* Top right* The four cow ribs used in the Compacted soil condition at Leintal Zoo.* Bottom left* Baited hole in the Loose soil condition.* Bottom right* Toto (hand-reared male, 37 years old) in the first bone excavating event recorded during session 2 of the Compacted soil condition at Leintal Zoo

At Leintal Zoo, we provided the chimpanzees with four disarticulated, defleshed, and simmered cow (*Bos taurus*) ribs (mean superoinferior length ± SD, 30.2 ± 1.19 cm; mean maximum width ± SD, 4.02 ± 1.04 cm; mean weight ± SD, 125.75 ± 36.26 g) cut at the backbone (Fig. 1). The number of ribs provided to the chimpanzees was limited to four because these were the specimens available at the local butcher shop at the time of the experiment. Ribs were chosen instead of limb bones given the results of the experiment at Kristiansand Zoo, which we hypothesized could be related to the length of the bones, as length has been previously shown to influence excavating tool selection in chimpanzees (Motes-Rodrigo et al. 2019). Before each session of the Compacted soil condition, we placed the four ribs in the study area next to the holes and flat on the ground. Ribs were recovered from the enclosure each day.

All bone specimens provided to the chimpanzees in both zoos were simmered for 90 min at 65 °C in order to disinfect them before their introduction into the chimpanzee enclosures. Once simmered, any remaining soft tissues were removed manually from the bones and they were then measured.

Chimpanzees participated in the experiment as a group. Recordings were made every day using one fixed camera (Sony HDR-CX330E Handycam) at Kristiansand Zoo and two cameras at Leintal Zoo.

### Coding

In each session we coded all interactions between the chimpanzees and the bone specimens. An ‘event’ was defined as each instance of a tool-related behaviour starting the moment a chimpanzee held a tool and ending when the chimpanzee stopped performing the behaviour for more than 3 s, changed behaviour or dropped the tool (Motes-Rodrigo et al. 2019). Tool excavating events started when the chimpanzee introduced any part of a tool into a hole and ended when the chimpanzee extracted the tool from the hole. If the tool was abandoned inside the hole, the tool excavating event ended when the chimpanzee released the tool. Individual chimpanzees were identified from video recordings. A second coder unfamiliar with the aims of the experiments independently coded all bone tool excavating behaviours using the ethogram provided in Table 2. Cohen’s κ (Cohen 1988) was calculated using the function kappa2 from the R package irr (Gamer et al. 2019).

## Results

### Kristiansand Zoo

The chimpanzees at Kristiansand Zoo were not observed excavating with the bones. However, manual excavation was observed during both the Loose soil condition (*n* = 12) and the Compacted soil condition (*n* = 119), and excavation using stick tools was observed during the Compacted soil condition (*n* = 34). Tool excavating events involved the use of the flags’ skewers in 23 cases and of sticks in ten cases, and always took place in combination with manual excavation (mean event duration ± SD, 50.5 ± 82.2 s, *n* = 131). In one case (which is not clear from the video recordings) it seems that one male (13 years old) used a stone to excavate [see the video in the Open Science Framework (OSF) folder]. The sticks used  to excavate were brought into the study area by the chimpanzees (solely based on the video recordings, it is not possible to determine from where). Seven of the nine individuals excavated at least once manually, and tool excavation was observed in three individuals (two males and one female). Food obtention was observed in 26 excavation events (two of which involved the use of tools). The chimpanzees were observed to perforate (*n* = 11) and probe (*n* = 18) using the tools. In five events the type of excavating behaviour could not be identified.

Despite the fact that the chimpanzees did not use the provided bones to excavate, they did interact with the bones in multiple other ways (Table 1). For example, chimpanzees were observed twice hitting bone specimens forcefully and repeatedly against a hard substrate (percussion), which led to the detachment of small bone fragments or to the fracture of the bone (see Fig. S1, Online Resource). One adult male individual was observed on several occasions using a skewer to pick and consume organic material from the medullary cavity (see video in OSF). Bone throwing was observed when, on two separate occasions, two males (13 and 37 years old) threw a bone towards the camera across the moat while standing bipedally and showing piloerection as part of a display towards an unfamiliar human present at the observation point from which the behavioural recordings were made (see videos in OSF). Unfortunately, given that many of these interactions took place in the indoor enclosure (the chimpanzees transported the bones there) during the time the study area was being filmed, there are no recordings available for most of them.

**Table 1:** Ethogram of bone-related behavioural forms observed at Kristiansand Zoo

Behaviour	Description	*n*
Hold	A chimpanzee holds the bone with one hand or one foot	7
Monopolization	A chimpanzee takes the bone and places it close to his/her body while engaged in another activity such as feeding or resting. If another individual tries to take the bone, the owner places it closer to his/her body or out of reach of the other individual	30^a^
Mouth	A chimpanzee puts the bone inside its mouth	8
Percussion	Holding the bone with one hand and in a sitting position, the chimpanzee hits a hard substrate (concrete) with it, fracturing the impacted surface of the bone	2
Play	A chimpanzee places the bone on the ground and, whilst sitting or standing, moves it along the substrate pressing it against the ground (like a toy car)	1
Sniff	A chimpanzee brings the bone close to its nose and sniffs it	14
Throw	A chimpanzee holding the bone with one hand and in bipedal position, moves its arm backwards and then quickly forwards, throwing the bone into the moat	2
Tool-assisted marrow consumption	Holding the bone with one hand, a chimpanzee uses a skewer gripped in the other hand to perforate the exposed spongy bone of the medullary cavity either longitudinally or from one side, perpendicular to the longitudinal axis. Then the skewer is retrieved, licked and the material ingested (see video in the OSF folder)	3
Transport	The bone is carried from one place to another in one of various ways: (1) in the mouth, while pressing the middle part of the bone with the lips; (2) while inserting one end of the bone into the oral cavity; or (3) while holding the bone with its foot or the hand that is still being used as support for locomotion	39

*n* Number of observations

^a^The exact number of observations was not recorded, thus *n* is an approximation

### Leintal Zoo

#### Bone excavation events and bone-related behavioural responses

The chimpanzees at Leintal Zoo performed 180 behavioural responses that involved the use of the cow ribs. Of these, 22 were bone-assisted excavating events (Table 2; Table S2, Online Resource). The non-excavating behavioural responses were classified as exploratory (133 events including sniff or mouth, see Table 1), social (display, eight events; hitting another chimpanzee, one event), and transport (16 events) (Table 2; Table S1, Online Resource).

**Table 2:** Ethogram of bone-related behavioural responses observed at Leintal Zoo

Behaviour	Description	*n*
Attempt to hit another chimpanzee	A chimpanzee tries to hit another chimpanzee with a bone	1
Dig	A chimpanzee holds a bone tool with one or both of its hands and places it on the ground. Then, while pressing the tool into the ground, moves it towards the edge of the hole, which leads to the extraction of soil from the hole	2
Display	A chimpanzee uses the bone as part of a display, hitting the ground with it or shaking it in the air, which causes the chimpanzees around him/her to move away	8
Hold	A chimpanzee holds the bone with its hand or its foot	63
Mouth	A chimpanzee puts the bone inside its mouth	11
Perforate	A chimpanzee inserts a tool into the ground perpendicularly and applies force by pushing the end of the bone further into the ground with one or more of its extremities. A power grip is used if one of the hands is holding the midsection of the bone. The bone is then retrieved and the end that had been inserted into the ground may or may not be visually and olfactorily inspected. No soil is displaced from the hole	4
Pound	A chimpanzee holds the bone with one or both of its hands and with powerful up and down movements stabs the ground repeatedly with the tool. No soil is displaced from the hole	3
Probe	A chimpanzee holds one end of a bone and places the other end in a hole while exerting light pressure or performing small wrist movements. The bone is then withdrawn and the inserted end may or may not be visually and olfactorily inspected. No soil is displaced from the hole	13
Sniff	A chimpanzee brings the bone close to its nose and sniffs it	48
Transport	The bone is carried from one place to another in one of several ways: (1) in the mouth, while pressing the middle part of the bone with the lips; (2) by inserting one end of the bone into the oral cavity; or (3) while holding the bone with a foot or the hand that is still being used as support for locomotion	16

Excavating behaviours are defined following Motes-Rodrigo et al. (2019)

Inter-observer reliability regarding bone excavating behaviours was found to be substantial (80% agreement, *k* = 0.63). The first bone excavating event was observed on 5 October 2018 and was performed by Toto (human-reared, male, 37 years old), who engaged in bone probing (for 2 s) for the first time at 00:02:54 hours in session 2 of the Compacted soil condition before proceeding to excavate manually (Fig. 1, bottom right panel; Table S2, Online Resource). Also in session 2, Toto performed for the first time bone perforating and bone pounding. By session 12, eight different individuals (two human reared and six mother reared) had manipulated the bones in the context of excavation (Table S2, Online Resource). The six mother-reared individuals that used bones as tools only performed bone probe (Table S2, Online Resource), while all other bone excavating events other than probe were performed by Toto (seven events—dig, pound, perforate) and Panya (human-reared, female, 11 years old; two events—pound, dig).

The bone excavation events lasted on average 6.45 ± 5.28 s (range 1–26 s). None of the bone excavating events led by itself to the retrieval of the buried food items. In some cases (*n* = 16), the food had already been dug out by another individual before excavation with bones occurred. In the cases where food was still present (*n* = 2), the food reward was not obtained as a direct consequence of using a bone. Instead, the apples were always obtained by manual digging, but the use of bones seemingly helped the chimpanzees in some cases to obtain the apples by loosening the soil and thus facilitating subsequent manual excavation. In 16 bone excavating events it was possible to determine in which part of the excavation sequence bones were used. Bone excavation in a given hole occurred before (*n* = 4), after (*n* = 8) or between (*n* = 4) manual excavation events in that same hole by a given individual.

### Behavioural responses to bones left inside holes

On seven occasions, bones were left inside the holes (with one end of the bone at the bottom of the hole). On one of these occasions, the same individual (Panya) reused the bone (13 s after leaving the bone in the first place). On six of these occasions, a different individual from the one who left the bone in the hole retrieved the bone. Only one individual (Panya) used a bone found inside a hole for excavating; this behaviour was observed on two occasions (session 2). In two other cases the arriving chimpanzee took the bone out of the hole, held it, sniffed it, and put it on the ground outside the hole (once in session 1, once in session 2). On the three remaining occasions (sessions 1, 8 and 12), the bone was taken out of the hole and placed on the ground without the chimpanzee previously performing any other action.

## Discussion

Our study reports, to the best of our knowledge, the first descriptions of the behavioural responses of captive chimpanzees to disarticulated bones in the context of food excavation. Given the various differences in group characteristics and testing protocols between the experiments conducted with each of the two chimpanzee groups, no direct comparisons of the chimpanzees’ behavioural responses towards the bones can be made between groups. Instead, we discuss the different factors that could have influenced the bone-related behaviours the chimpanzees exhibited in each of the two populations separately.

### Kristiansand Zoo

We did not observe bone excavation in the Kristiansand chimpanzees. Instead, they obtained the buried food rewards both manually and by using plant tools. The absence of bone excavation in the Kristiansand chimpanzee population could have multiple, non-exclusive explanations. In a previous study of plant tool excavation conducted with this same population, we found that tool length was the only tool characteristic that influenced the probability that the chimpanzees used a stick as an excavating tool (Motes-Rodrigo et al. 2019). The length range of the sticks selected and used as excavating tools in our previous study was 28–57.5 cm [Table 5 in Motes-Rodrigo et al. (2019)]. The length of the bones provided to the Kristiansand chimpanzees in the present study was outside this range (7.3–22.6 cm). Therefore, it is possible that the bones provided were not perceived as suitable for the task due to their physical dimensions, as they were shorter than the plant tools the chimpanzees use to excavate.

The lack of bone tool use in this population could have also been related to the fact that the epiphysis of the bones had been removed and some of the bones had been split open along their long axis, exposing the cancellous tissue and the medullary cavity. Although we simmered the bones and dissolved part of the marrow before giving them to the chimpanzees, some marrow remained in the medullary cavity. The exposure of the endosteal bone surfaces, with adhering organic material (e.g. small quantities of fat and meat within the spongy bone), might have distracted the chimpanzees from using the bones as excavating tools. Indeed, we observed tool-assisted bone consumption in Kristiansand Zoo by one individual, indicating that the chimpanzee perceived the bones as a food source, similarly to what has been reported in the wild (Boesch and Boesch 1990; Sanz and Morgan 2007). However, although we observed bone consumption, we could not determine from the video recordings whether the chimpanzee consumed the bone marrow itself or other organic materials within the medullary cavity.

The fact that the Kristiansand chimpanzees often engaged in successful manual and stick tool excavation both during a previous study and the present one, might have made the use of bones as excavating tools unnecessary. Based on the relative profitability hypothesis (Rutz and St Clair 2012), the emergence of certain types of tool use can be related to the relative advantage that a particular form of tool use poses compared to not using a tool or using another form of tool use. Therefore, it is possible that using the bones as tools in this population did not constitute a sufficient improvement compared to using plant tools and/or manual excavation. Despite not engaging in bone excavation, the Kristiansand chimpanzees performed other behaviours, including using bones to hit the concrete floor of the indoor enclosure.

### Leintal Zoo

We found that some of the Leintal chimpanzees used cow ribs as tools during excavation bouts, although these chimpanzees had never been exposed to bones before and had only extremely limited experience with underground food items (i.e. naturally occurring insects and a buried juice bottle). These observations of bone-assisted excavation involved several actions similar to those described for the Kristiansand population in the context of stick tool excavation (Motes-Rodrigo et al. 2019) and consisted of fairly short events (*n* = 22; median = 5; mean ± SD, 6.45 ± 5.28 s). Our results suggest that the use of bones as tools by the Leintal chimpanzees was opportunistic rather than necessary. The fact that bone excavation never took place independently of manual excavation and that the use of bones did not lead in itself to the retrieval of food suggests that the use of bones was a complement to manual excavation rather than a behaviour necessary to directly obtain the buried food items. The use of bones, therefore, does not seem to have provided a sufficient advantage over manual excavation for the chimpanzees to predominantly excavate using them [see also the relative profitability hypothesis of Rutz and St Clair (2012)]. This combination of tool-assisted and manual excavation was also described in bearded capuchin monkeys from Serra da Capivara National Park, who engage in stone-assisted excavation to obtain underground foods. During excavation bouts, individuals were reported to hit the ground with hand-held stones several times while manually scooping away soil before releasing the stone and proceeding to excavate manually (Moura and Lee 2004). More recent studies have confirmed that tool excavation in bearded capuchin monkeys is always accompanied by manual excavation (Falótico et al. 2017).

In the experiment at Leintal, the two chimpanzees that engaged in bone excavating behaviours other than probing had both spent time as infants in the care of humans. However, it seems unlikely that rearing conditions alone could explain the performance of these chimpanzees, as there were other human-reared chimpanzees in the group who did not use the bones to excavate even though they had access to the study area. Given that the Leintal chimpanzees were naïve to detached bones before the start of the experiment, we can conclude that at least the first chimpanzee that engaged in bone excavation individually learnt (or innovated) this behaviour. However, considering that, from the beginning, the chimpanzees were tested only in a group setting and that no complementary individual testing was conducted, it is difficult to draw conclusions about the specific social and/or individual learning mechanisms underlying the expression of bone tool use by the subsequent tool users at this stage (see also Bandini et al. 2021). Future studies could evaluate the learning mechanisms underlying the expression of bone tool use, as well as the strength of social transmission of this behaviour, by using stepwise testing and network-based approaches, respectively (e.g. Franz and Nunn 2009; Hoppitt et al. 2010; Bandini et al. 2021).

### Limitations

Our study might have been limited by the modest number of bones and excavating sites that we provided to each group of chimpanzees and the impossibility of performing a crossed-factor experimental design. The number of bones provided in Kristiansand Zoo was determined by the availability of privately owned horses donated to the zoo. In addition, low-ranking females of this population of chimpanzees often monopolized the bones, carrying them away from the study area once the males had left the excavation sites and the bones that had been placed there. This monopolization meant that the bones were in the study area for limited periods of time. However, the chimpanzees always had access to bones at the beginning of the Compacted soil condition sessions, meaning that although monopolization limited the time the bones were in the study area, it did not prevent their use. At Leintal Zoo, the number of cow ribs available was determined by the cow-butchering schedule and availability in the town where the experiments were conducted. The reduced number of holes at both sites also meant that not all individuals could participate in the task at the same time, and that high-ranking individuals occasionally monopolized the excavating sites, limiting the access of the remaining individuals to the study area and materials. This was especially true in Leintal, given its large group size. Furthermore, the lack of natural vegetation available to the Leintal population prevented the chimpanzees from having to choose between bones and sticks as excavating tools. Another limitation was that we were not able to make direct comparisons between chimpanzee groups in terms of their behavioural responses to the detached bones because it is not possible to determine which of the factors that varied between groups (e.g. material availability, bone characteristics or previous digging experience) are responsible for the observed variation. To overcome these limitations, future studies could employ identical protocols to test and compare multiple chimpanzee groups with the same repertoire of raw materials that could be used as tools (e.g. bones, sticks, plastic), as well as evaluate the effect of tool dimensions in tool selection by providing tools of the same material but with differing physical characteristics (see also McGrew 2021). Finally, replicating the present study using larger study areas with more excavating sites and over a longer experimental period would allow more individuals to engage in the task and for researchers to evaluate whether bone-related behaviours change or emerge over time.

### Conclusions

Our results represent some of the first observations on the spontaneous behavioural responses of captive chimpanzees towards bones (see also Pickering and Wallis 1997). In Kristiansand Zoo, bones were not used as excavating tools even though other bone-related behaviours were observed in this population. Our findings from the Leintal population show that chimpanzees have the ability to innovate the use of bones to assist in the excavation of buried food items employing similar tool excavating actions to those described in previous plant tool excavating studies (Motes-Rodrigo et al. 2019). We hope that our study fosters novel primate archaeological research investigating the emergence and potential transmission of bone-related behaviours, including bone tool use, in this species. Bone-use experiments with chimpanzees could be valuable in providing, for instance, reference collections of bone specimens linking behavioural actions and use-wear, which could help researchers to better interpret bone artifacts from the archaeological record [for similar approaches, see Arroyo et al. (2016, 2021)].

## Supplementary Information

Below is the link to the electronic supplementary material.

Supplementary file1 (DOCX 564 KB)

## Data Availability

Raw data and sample video clips can be found in the OSF link: https://osf.io/cewdz/?view_only=e9c48b3a63134e37a1f3032f5d7c74fa
