# Supplementary material for: Bone-related behaviours of captive chimpanzees (Pan troglodytes) during two excavating experiments
Source: Primates. 2022 Nov 19;64(1):35–46. doi: 10.1007/s10329-022-01033-w (PMC9842580; doi:10.1007/s10329-022-01033-w)
Supplement: Supplementary file 1 — Supplementary file1 (DOCX 564 KB) [file 10329_2022_1033_MOESM1_ESM.docx]

Bone-related behaviours of captive chimpanzees (*Pan troglodytes*) during two excavating experiments

Alba Motes-Rodrigo^a,b^, Claudio Tennie^a^ & R. Adriana Hernandez-Aguilar^b,c^

a. Department of Early Prehistory and Quaternary Ecology, University of Tübingen, 72070, Germany

b. Department of Ecology and Evolution, University of Lausanne, 1015, Switzerland

c. Department of Social Psychology and Quantitative Psychology, University of Barcelona, Serra Hunter Program, Barcelona, 08035, Spain

d. Centre for Ecological and Evolutionary Synthesis, University of Oslo, NO-0316, Norway

**Corresponding author:** AMR, albamotes7@gmail.com, ORCID: 0000-0002-4444-7723

**Supplementary materials & methods**

*Keeper reports*

The keepers at Leintal zoo reported that the chimpanzees had been observed several times, since the summer of 2018, "scratching soil" (as defined by the keepers: "using wooden sticks on the ground") in the area surrounding a climbing pole in the outdoor enclosure. The keepers speculated that the chimpanzees might have been looking for or feeding on insects, or simply playing with dirt (the keepers reported that it is a common behavior in this group to collect loose dirt by hand and throw it at other individuals, a behaviour also observed by the experimenter). However, no recordings of these episodes were available and no more detailed descriptions of the behavior could be obtained.

On one occasion in 2017, a plastic bottle filled with juice was buried by the keepers head up and without a cap in the outdoor enclosure of the chimpanzees to motivate them to dip in the juice with sticks. The keepers reported that after some time dipping sticks in the bottle and sucking the juice from the sticks the chimpanzees dug up the bottle (this was not measured so the duration of these events is not available). However, no recordings of how the bottle was extracted were made and therefore it is not possible to know if the chimpanzees used tools in the process or simply pulled up the bottle by gripping or biting its head. Crucially, all three keepers confirmed that none of the chimpanzees had ever had any previous experience with bones (nor bone tools).

The keepers at Kristiansand zoo confirmed that the chimpanzees had no previous experience with bones (or bone tools). The chimpanzees at Kristiansand zoo had previous experience in excavation (both manually and with tools) as a consequence of an excavating experiment conducted in 2013 and reported in Motes-Rodrigo et al. (2019).

Supplementary Table 1: Measurements and characteristics of the horse bones provided to the chimpanzees at Kristiansand zoo. Length refers to the maximum length of the longest axis of the bone. Max width refers to the maximum width of the bone. Columns 5 to 8 refer to the angle measurements of the four bone ends from which the epiphysis had been removed. These angles were measured with a goniometer by placing one arm on the periostal surface of the bone and the other arm on the sawed apical surface. Bone type refers to the bone from which the bone fragments were created. F means horse femur and H means horse humerus.

| Tool nr | Days of use | Length (cm) | Max width (cm) | Angle left top | Angle right top | Angle left bottom | Angle right bottom | Weight (g) | Bone type |
| --- | --- | --- | --- | --- | --- | --- | --- | --- | --- |
| 1 | 5 | 18.6 | 6.6 | 98 | 94 | 97 | 98 | 354 | F |
| 2 | 5 | 14.2 | 7,2* | 118 | 65 | 90 | 96 | 496 | F |
| 3 | 5 | 15 | 8 | 94 | 97 | 82 | 104 | 270 | F |
| 4 | 1 | 14.6 | 5.8 | 95 | 104 | 92 | 94 | 270 | F |
| 5 | 1 | 11.7 | 5.9 | 117 | 90 | 89 | 89 | 210 | F |
| 6 | 1 | 11.1 | 5.9 | 91 | 101 | 89 | 61 | 178 | F |
| 7 | 1 | 15.4 | 6 | 106 | 95 | 92 | 91 | 264 | F |
| 8 | 1 | 7.8 | 6.8 | 5.9* | 72 | 6.8* | 88 | 204 | H |
| 9 | 1 | 7.5 | 8.6 | 6.6* | 90 | 8.5* | 90 | 264 | H |
| 10 | 2 | 17 | 6.1 | 74 | 98 | 87 | 93 | 222 | H |
| 11 | NA | 16.3 | 5.2 | 95 | 96 | 76 | 95 | 276 | H |
| 12 | 2 | 18.1 | 5.5 | 93 | 100 | 79 | 94 | 290 | H |
| 13 | 2 | 18.5 | 5.6 | 100 | 98 | 93 | 80 | 320 | H |
| 14 | 1 | 19.5 | 6.8 | 115 | 83 | 82 | 90 | 340 | H |
| 15 | NA | 19.6 | 6.9 | 111 | 81 | 81 | 91 | 336 | H |
| 16 | 2 | 17.6 | 5 | 85 | 93 | 97 | 75 | 256 | H |
| 17 | NA | 17.6 | 5.4 | 96 | 89 | 76 | 97 | 416 | H |
| 18 | NA | 22.6 | 6.1 | 103 | 92 | 94 | 97 | 344 | H |
| 19 | NA | 23 | 5.8 | 92 | 105 | 82 | 95 | 560 | H |

Supplementary Table 2: Measurements and characteristics of the cow bones provided to the chimpanzees at Leintal zoo. Length refers to the maximum length of the longest axis of the bone. Max width refers to the maximum width of the bone.

| Tool nr | Days of use | Length (cm) | Max width (cm) | Weight (g) | Bone type |
| --- | --- | --- | --- | --- | --- |
| 1 | 6 | 31 | 5.5 | 176.3 | Rib |
| 2 | 6 | 30 | 3.7 | 120.2 | Rib |
| 3 | 6 | 31.2 | 4.5 | 116.4 | Rib |
| 4 | 6 | 28.6 | 3.1 | 90.1 | Rib |

**Supplementary results**

Supplementary Table 3: Bone-excavating events recorded. Order indicates the use of bones relative to manual excavation (before: before excavating manually; after: after excavating manually; between: between manual excavation bouts). L= left hand, B =both hands, F=foot, R=right hand, RF=right foot, LF=left foot. Unk stands for unknown and indicates that the information could not be coded from the videos.

| **Session** | **Date** | **Time of  day** | **Start** | **Duration (sec)** | **Individual** | **Rearing history** | **Action** | **Limb** | **Order** | **Apple present** |
| --- | --- | --- | --- | --- | --- | --- | --- | --- | --- | --- |
| 2 | 5.10.18 | afternoon | 00:02:54 | 2 | Toto | human | probe | L | before | 1 |
| 2 | 5.10.18 | afternoon | 00:05:15 | 8 | Donald | mother | probe | L | unk | unk |
| 2 | 5.10.18 | afternoon | 00:08:05 | 4 | Toto | human | pound | R | after | 0 |
| 2 | 5.10.18 | afternoon | 00:09:01 | 10 | Toto | human | perforate | B,LF | before | 0 |
| 2 | 5.10.18 | afternoon | 00:10:43 | 12 | Toto | human | perforate | B,LF | after | 0 |
| 2 | 5.10.18 | afternoon | 01:06:42 | 2 | Girlie | mother | probe | L | after | 0 |
| 2 | 5.10.18 | afternoon | 00:00:08 | 2 | Panya | human | probe | L | unk | 0 |
| 2 | 5.10.18 | afternoon | 00:00:11 | 9 | Panya | human | probe | L | unk | 0 |
| 2 | 5.10.18 | afternoon | 00:01:11 | 1 | Zicklein | mother | probe | R | unk | 0 |
| 2 | 5.10.18 | afternoon | 00:02:07 | 5 | Panya | human | pound | R | before | 0 |
| 2 | 5.10.18 | afternoon | 00:02:12 | 9 | Panya | human | dig | R | before | 0 |
| 2 | 5.10.18 | afternoon | 00:03:08 | 4 | Panya | human | probe | L | between | 0 |
| 6 | 7.10.18 | afternoon | 00:00:00 | 8 | Toto | human | perforate | RF | unk | 0 |
| 6 | 7.10.18 | afternoon | 00:00:09 | 4 | Toto | human | pound | R | unk | 0 |
| 7 | 8.10.18 | morning | 00:29:01 | 8 | Garibal | mother | probe | R | after | 0 |
| 8 | 8.10.18 | afternoon | 01:32:22 | 5 | Toto | human | probe | R | after | unk |
| 8 | 8.10.18 | afternoon | 01:37:50 | 4 | Girlie | mother | probe | L | after | unk |
| 8 | 8.10.18 | afternoon | 01:38:20 | 6 | Toto | human | perforate | LF | after | unk |
| 10 | 9.10.18 | afternoon | 01:26:57 | 5 | Toto | human | dig | R | between | 1 |
| 10 | 9.10.18 | afternoon | 01:36:30 | 26 | Lutz | mother | probe | B | between | 0 |
| 12 | 10.10.18 | afternoon | 02:22:26 | 2 | Donald | mother | probe | L | after | 0 |
| 12 | 10.10.18 | afternoon | 02:24:07 | 6 | Schon | mother | probe | L | between | 0 |


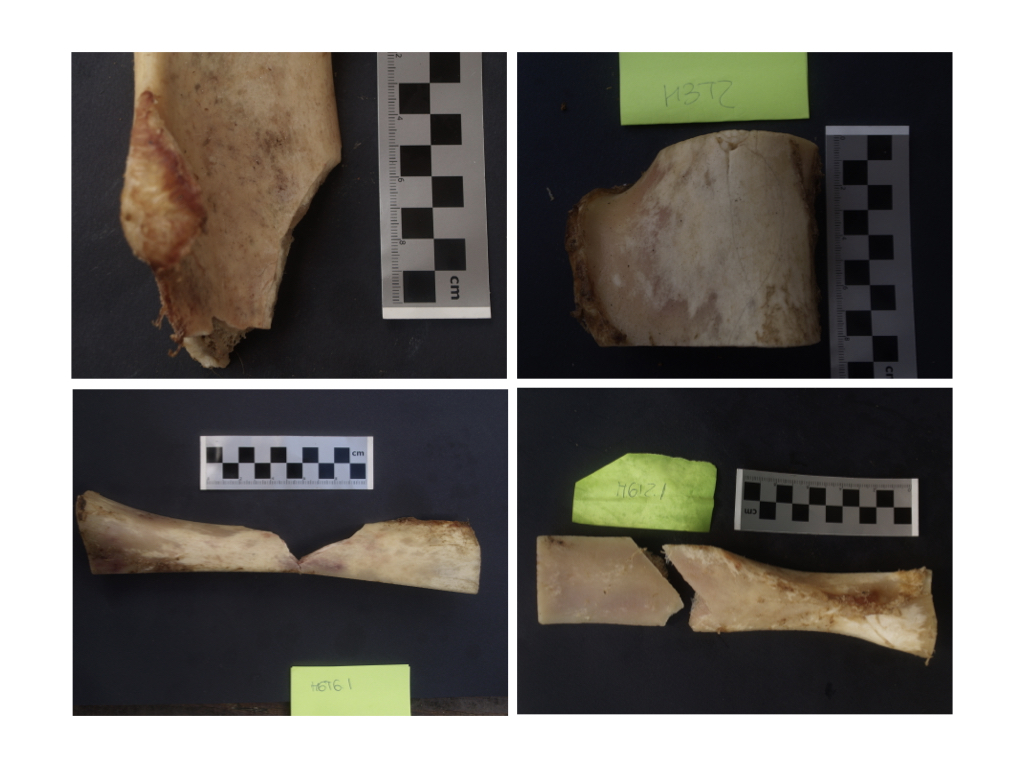


Supplementary Figure 1: Four examples of fragmented bones that were recovered from the chimpanzee enclosures.
